# Microcirculatory blood flow is related to clinical signs of impaired organ perfusion, and its dynamics to the macrohemodynamic concept of fluid responsiveness

**DOI:** 10.1186/cc10844

**Published:** 2012-03-20

**Authors:** A Pranskunas, M Koopmans, V Pilvinis, P Koetsier, EC Boerma

**Affiliations:** 1 Lithuanian University of Health Sciences, Kaunas, Lithuania; 2 Medical Centre Leeuwarden, the Netherlands; 3 Hospital of Lithuanian University of Health Sciences, Kaunas, Lithuania

## Introduction

Fluid responsiveness is not equal to a clinical need for fluid therapy. The aim of our study was to assess the incidence of microcirculatory flow alterations, according to a predefined arbitrary cut-off value, in patients with clinical signs of impaired organ perfusion. The secondary endpoint was to establish the correlation between the microcirculatory and macrocirculatory response to a fluid challenge.

## Methods

We performed a prospective, single-centre, observational study. Included were ICU patients ≥18 years with invasive hemo-dynamic monitoring and clinical signs of impaired organ perfusion, as the principal reason for fluid administration. Fluid challenge was performed by the infusion of 500 ml crystalloid or a balanced colloid (Volulyt^®^) solution in 30 minutes. Before and after fluid challenge, systemic hemodynamics and direct *in vivo *observation of the microcirculation were obtained with sidestream dark-field imaging. Assessment of microcirculatory parameters of convective oxygen transport (microvascular flow index (MFI) and proportion of perfused vessels), and diffusion distance (perfused vessel density and total vessel density) was done using a semiquantitative method.

## Results

We enrolled 50 patients. MFI <2.6 was present in 66% of the patients. After fluid challenge, signs of impaired organ perfusion reduced from 100% to 68% of the patients, *P *< 0.001. The incidence of MFI <2.6 decreased to 46%, and was higher in patients with persistent signs of impaired organ perfusion: 56% versus 25%, *P *= 0.04. Median MFI increased from 2.5 (2.3 to 2.8) at baseline to 2.7 (2.4 to 2.8) after fluid challenge, *P *= 0.003, but its change was only significant in fluid-responsive patients.

## Conclusion

These data demonstrate a relationship between clinical signs of impaired organ perfusion and MFI <2.6. Fluid responsiveness did not discriminate between patients with and without clinical signs of impaired organ perfusion or MFI <2.6. However, significant improvement of microvascular alterations and attenuation of clinical signs of impaired organ perfusion was restricted to patients who were fluid responsive. Noninvasive assessment of microvascular perfusion may help to define patients with potential need for fluid therapy, and to evaluate its effect.

